# Immune-iron homeostasis deciphers resistance divergence to *Riemerella anatipestifer* in ducks

**DOI:** 10.3389/fmicb.2025.1627631

**Published:** 2025-07-11

**Authors:** Mengyun Zou, Xiaolan Xiong, Weihong Zhang, Yue Wei, Weiguo Tang, Qipeng Wei, Cong Huang, Zhaofeng Kang, Jinfang Xie, Jiangnan Huang

**Affiliations:** Institute of Animal Husbandry and Veterinary Medicine, Jiangxi Academy of Agricultural Sciences, Nanchang, China

**Keywords:** duck, *Riemerella anatipestifer*, disease resistance, immune response, iron metabolism

## Abstract

*Riemerella anatipestifer* (RA) causes substantial economic losses in global waterfowl production. This study compared immune responses and iron metabolism of ducks against RA infection between commercial White Kaiya ducks (WK) and native Ji’an Red-feathered ducks (JR), and between resistant (R) and susceptible (S) individuals within each breed. Results revealed JR ducks exhibited lower morbidity (85.63% vs. 100%) and mortality (68.75% vs. 88.76%) than WK ducks under identical infection conditions. Susceptible individuals in both breeds showed severe weight loss, splenomegaly, and multiorgan histopathological damage. Conversely, phenotype-specific resilience differed by breed: R-JR ducks maintained stable body weight, normal spleen indices, and minimal lesions versus weight loss, splenomegaly, and pathological persistence in R-WK ducks. Furthermore, S-JR ducks exhibited elevated tumor necrosis factor-*α* (TNF-*α*) with down-regulated IgA, interferon-*γ* (IFN-γ), interleukin-22 (IL-22), IL-4, and IL-10 whereas R-JR ducks maintained stable IgA/Th1/Th2 cytokines with elevated IL-17A. In contrast, S-WK ducks exhibited elevated IgM/IgG/TNF-*α* with static IgA levels, and decreased IL-17A, while R-WK ducks showed lower IgM with stable IgG/Th1/Th2/Th17 level S relative to S-WK ducks. Furthermore, pathological iron accumulation occurred in all infected WK ducks but not resistant JR ducks. Collectively, these breed-specific differentials highlight JR ducks’ coordinated immune-iron homeostasis versus WK ducks’ cytokine and iron dysregulation, providing insights for disease-resistant breeding strategies.

## 1 Introduction

With the global shift toward healthier dietary patterns, the global meat consumption structure has shifted toward poultry. According to statistical data from China’s Waterfowl Industry Technology System, duck meat—renowned for its low-fat, high-protein profile—has emerged as a preferred choice, positioning ducks as the third most consumed meat species in China after pork and chicken (Hou and Liu, 2025). However, the sustainable development of duck production is severely constrained by *Riemerella anatipestifer* (RA)-induced infectious serositis, a devastating disease characterized by systemic fibrinous exudation in vital organs (pericardium, hepatic capsule, and meninges). Epidemiological data reveal alarming infection rates of 90% and co-infection mortality approaching 100%, causing massive production and economic losses to the waterfowl industry (Chen et al., 2024). RA infection leads to growth retardation, immune dysfunction, impaired production performance, and reduced feed conversion efficiency, causing huge economic losses to the waterfowl industry (Subramaniam et al., 2000; Zhu et al., 2017; Zhang et al., 2024). Currently, vaccination and antibiotics are effective means of controlling RA infection. Nevertheless, due to the antigenic diversity of RA and the emergence of multidrug-resistant RA strains, the existing vaccines and antibiotics have limitations (Lyu et al., 2023; Liu J. et al., 2024; Yang et al., 2024; Zhang et al., 2024).

Disease-resistant breeding has emerged as a promising strategy for combating poultry infections, evidenced by successful application against duck hepatitis A virus and Salmonella infections (Wang et al., 2017; Dar et al., 2022; Wang et al., 2023). Notably, poultry disease resistance exhibits moderate heritability (h^2^ = 0.2–0.4) with notable breed-specific variability, showing significant differences among varieties and individuals (Wang et al., 2017; Li et al., 2018; Hako Touko et al., 2021; Miyumo et al., 2023). For example, Wang et al. (2017) demonstrated host differences influencing the Pekin duck flock’s resistance and susceptibility to Duck Hepatitis A Virus Genotype 3 (Wang et al., 2017), and our previous studies revealed that indigenous poultry breeds (e.g., Qingjiao Ma chicken) display superior resistance to Mycoplasma infections compared to commercial lines (Li et al., 2025). This paradigm extends to RA susceptibility: fast-growing commercial ducks (Peking, Cherry Valley, Muscovy) show heightened vulnerability, while indigenous breeds exhibit robust resistance (Li et al., 2024). Therefore, in-depth research on the regulatory mechanisms influencing the resistance of different duck breeds to RA are of great significance for controlling RA infection.

Host defense against RA infection involves a coordinated interplay between innate and adaptive immune pathways. During the initial phase of infection, RA invasion triggers the activation of innate immunity, characterized by rapid recruitment of macrophages and dendritic cells, along with pro-inflammatory cytokine cascades (Cammayo et al., 2020). This inflammatory microenvironment primes subsequent adaptive immune responses, marked by the sequential production of pathogen-specific IgM and IgG antibodies (Kang et al., 2018; Eladl et al., 2023). Notably, resistant ducks exhibited distinct immune signatures during RA infection, including significant upregulation of Th1/Th2 cytokines such as interleukin-2 (IL-2), IL-6, IL-8, and interferon-*γ* (IFN-*γ*), coupled with synergistic elevation of immunoglobulins (IgM/IgG) (Wang et al., 2017). Iron, primarily stored in the spleen and liver, plays a crucial regulatory role in host resistance to microbial pathogens (Spiga et al., 2023). The host restricts pathogen access to iron—a strategy known as “nutritional immunity”—to combat infections (Cassat and Skaar, 2013; Nairz et al., 2014). As a home synthesis-deficient bacterium, RA’s growth and pathogenicity depend entirely on host iron sources (Liu M. et al., 2024; Wang et al., 2024; Wang et al., 2025). Previous studies have shown that RA infection disrupts iron metabolism gene expression in duckling livers (Zhou et al., 2013). Therefore, we hypothesize that host resistance to RA infection is associated with cytokine-mediated immune responses, antibody kinetics, and strategic iron restriction (e.g., liver/spleen iron isolation).

The Ji’an Red-feathered Duck (JR), an indigenous breed with genetic stability and disease resilience, and the White Kaiya (WK) duck, a commercial breed optimized for growth performance, provide an ideal model to test this hypothesis. Hence, this study will horizontally compare the two breeds’ resistance to RA infection, their cytokine expression profiles, and iron metabolism level changes, and vertically compare the resistance of different individuals within the same breed, aiming to reveal the expression and role of cytokine immune-iron metabolism in ducks’ resistance to RA infection. This study will contribute to revealing the molecular mechanism of RA resistance in local ducks and provide theoretical basis and genetic resources for breeding new Ra-resistant varieties.

## 2 Materials and methods

### 2.1 Ethics in animal experimentation

All animal experiments were approved by the Experimental Animal Ethics Committee of the Institute of Animal Husbandry and Veterinary Medicine, Jiangxi Academy of Agricultural Sciences (approval number: 2024-JXAAS-XM-05). The experimental procedures strictly adhered to the International Guiding Principles for Biomedical Research Involving Animals issued by the Council for the International Organizations of Medical Sciences. Before the experiments, the animals were acclimatized to the experimental environment for a week to ensure their well-being. The housing conditions were maintained at a temperature of (25 ± 2)°C, relative humidity of (55 ± 5)%, and a 12-h light/dark cycle.

### 2.2 RA strain and culture

The RA-YM strain was kindly donated by the State Key Laboratory of Agricultural Microbiology at Huazhong Agricultural University (Wuhan, China). As previously methods (Wang et al., 2024), RA-YM strain was inoculated on TSA solid medium with 5% calf serum and cultured at 37°C for 36 h at 5% CO_2_. A single colony was selected and placed in 10 mL TSB liquid medium and incubated at 37°C with shaking until an exponential growth phase was achieved (Zhou et al., 2013). The final challenge inoculum concentrations were determined by plating 0.1 mL of a 10-fold serial dilution onto Tryptic Soy agar containing 5% newborn calf serum.

### 2.3 Animal experiments

For this study, a local Chinese duck breed, the Ji’an Red-feather duck (JR), and a commercial meat duck breed, the White Kaiya duck (WK), were selected. A total of 208 one-day-old healthy WK ducks (*Anas platyrhynchos*) and 222 one-day-old healthy JR ducks, all with no prior exposure to RA, were obtained from a Ji’an National Breeding Ground in Ji’an, China. These ducks were randomly allocated into two primary groups: the non-infected control group and the infected group. At 4-week-old, the ducks in the infected group were inoculated with 0.5 mL of a suspension containing 1.0 × 10^6^ colony-forming units (CFU) of RA-YM strian via the footpad. The control group received an equal volume of sterile phosphate-buffered saline (PBS) using the same injection method. After inoculation, the clinical symptoms of the infected ducks were carefully monitored and recorded every 6 h. Based on the observed clinical symptoms, onset patterns, and mortality rates, 72 h post-infection (hpi) was determined as the key time point for distinguishing between susceptible and disease-resistant individuals within breed. Ducks that remained clinically asymptomatic at 72 hpi were classified as the disease-resistant group (R-group), whereas those exhibiting terminal symptoms were categorized as the susceptible group (S-group). In accordance with approved ethical procedures, all ducks were humanely euthanized by cervical dislocation at 72 hpi. Subsequently, body weight was measured, and the spleen index was calculated. Gross pathological observations were made and documented. The brain, heart, liver, and spleen tissues from both infected and control ducks were collected and immediately immersed in 4% polyformaldehyde for further analysis.

### 2.4 ELISA assay

Serum samples were collected from ducks in each group. The levels of IgA (RX700259D/2024, ruixinbio, China), IgM (RX700194D/2024, ruixinbio, China), IgG (RX700258D/2024, ruixinbio, China), IL-17A (RX700403D/2024, ruixinbio, China), TNF-*α* (RX700248D/2024, ruixinbio, China), IL-22 (RX700407D/2024, ruixinbio, China), IFN-*γ* (RX700274D/2024, ruixinbio, China), IL-4 (RX700217D/2024, ruixinbio, China), IL-6 (RX700270D/2024, ruixinbio, China), IL-10 (RX700216D/2024, ruixinbio, China), and GPX4 (MM-1005D1/202402, ruixinbio, China) were measured using commercially available ELISA kits. The absorbance value was detected at 450 nm using an automatic microplate reader (Bio-Rad, Hercules, CA, USA). Each group (Control-group, R-group, S-group) had at least five samples, and the mean value was calculated for statistical analysis.

### 2.5 Serum iron determination

Serum samples were collected from ducks in each group, each group had at least five samples. The level of serum iron was evaluated via Serum Iron determination kit (colorimetry) (Nanjing Jiancheng Biology, Nanjing, China). The absorbance value was detected at 520 nm using an automatic microplate reader (Bio-Rad, Hercules, CA, USA). Each group had at least nine duplicates, and the mean value was calculated for statistical analysis.

### 2.6 Histopathological examination

The brain, liver, heart, and spleen tissues of ducks from both the infected and non-infected groups were subjected to histopathological evaluation using hematoxylin–eosin (H&E) staining. The tissues were excised and fixed with 10% neutral buffered formalin for 24 h. After ethanol and dimethylbenzene processed processing, the tissues were embedded in paraffin, sliced (Leica, LEICARM2245, Germany), and then stained with H&E (ServiceBio, China). Finally, pathological changes were observed using a light microscope (Olympus, BX53, Japan). Each group had at least three samples.

### 2.7 Prussian blue dyeing assay

The DAB+PB Prussian blue staining process is as follows: First, fix the brain, liver, heart, and spleen tissues from both the infected and non-infected groups with 4% paraformaldehyde at 4°C for 24 h, and then wash them with PBS. Determine whether to perform antigen retrieval according to the nature of the antigen. Subsequently, incubate with specific primary antibodies at 4°C overnight, and then incubate with secondary antibodies (HRP-conjugated) at room temperature for 1–2 h, and add DAB substrate for color development. After that, perform Prussian blue staining with a mixture of potassium ferrocyanide and hydrochloric acid in the dark at room temperature for 30–60 min, and then counter - stain with nuclear fast red for 5–10 min. Finally, dehydrate, clear, mount the slides, and conduct microscopic examination. Positive and total cells were counted using ImageJ software. Each group had at least three samples.

### 2.8 Terminal deoxynucleotidyl transferase-mediated dUTP nick end labeling (TUNEL) analysis

To analyze RA-induced apoptosis in duck tissues, a TUNEL assay was performed using a kit purchased from Servicebio Biotechnology Co., Ltd. (Wuhan, China). The procedure strictly adhered to the manufacturer’s instructions provided with the kit. Tissue sections were dewaxed in xylene and rehydrated with a gradient of ethanol solutions, then repaired with Proteinase K and permeabilized with Triton X-100. The TUNEL reaction solution was added and incubated at 37°C in the dark for 60 min, followed by nuclear staining with DAPI. After mounting with an anti-fluorescence quenching agent, images were captured at ×40 magnification in randomly selected fields under a fluorescence microscope. Positive and total cells were counted using ImageJ software, and the apoptosis rate was calculated as the ratio of the two. Each group had at least three samples.

### 2.9 Statistical analysis

IBM SPSS Statistics 19 software (Armonk, New York, USA) was utilized for data analysis. The differences between and within groups were analyzed by one-way ANOVA followed by Duncan’s multiple-range test. The data are presented as the mean ± standard deviation (SD). Each group consisted of at least three biological replicates. A value of *p* < 0.05 was considered statistically significant.

## 3 Results

### 3.1 Establishment of RA-infected duck models

Two genetically distinct duck breeds (JR and WK) were challenged with a virulent RA strain via intravenous injection through the flipper vein to establish infection models. Clinical observations revealed progressive symptom development: at 6 h post-infection (hpi), both JR and WK ducklings developed primary clinical signs characterized by lethargy, impaired locomotor activity, and appetite suppression. By 12 hpi, disease advancement was evidenced by severe postural instability manifesting as inability to maintain standing position, incipient paralysis, labored respiration, and complete feed refusal, with initial mortality events recorded at this stage. At 24 hpi, advanced symptoms emerged, characterized by yellowish-green diarrhea and neurological manifestations such as opisthotonos (abnormal arched-back posture) and generalized tremors. The infection demonstrated acute progression with mortality onset at 12 hpi and reaching peak between 48 and 72 hpi, consistent with acute infection characteristics. Comparative analysis revealed significant interbreed differences (Table 1): WK ducks exhibited 100% morbidity and 88.76% mortality rates, while JR ducks demonstrated markedly lower rates of 85.63 and 68.75%, respectively (Table 1).

**Table 1:** Morbidity and mortality of RA-infected ducks.

Breed	Infected ducks	The number of deaths after infection						Morbidity	Mortality
		12 hpi	24 hpi	48 hpi	72 hpi	96 hpi	120 hpi		
JR duck	192	1	17	55	49	10	0	85.63%	68.75%
WK duck	178	1	22	48	72	7	8	100%	88.76%

**Figure 1:**
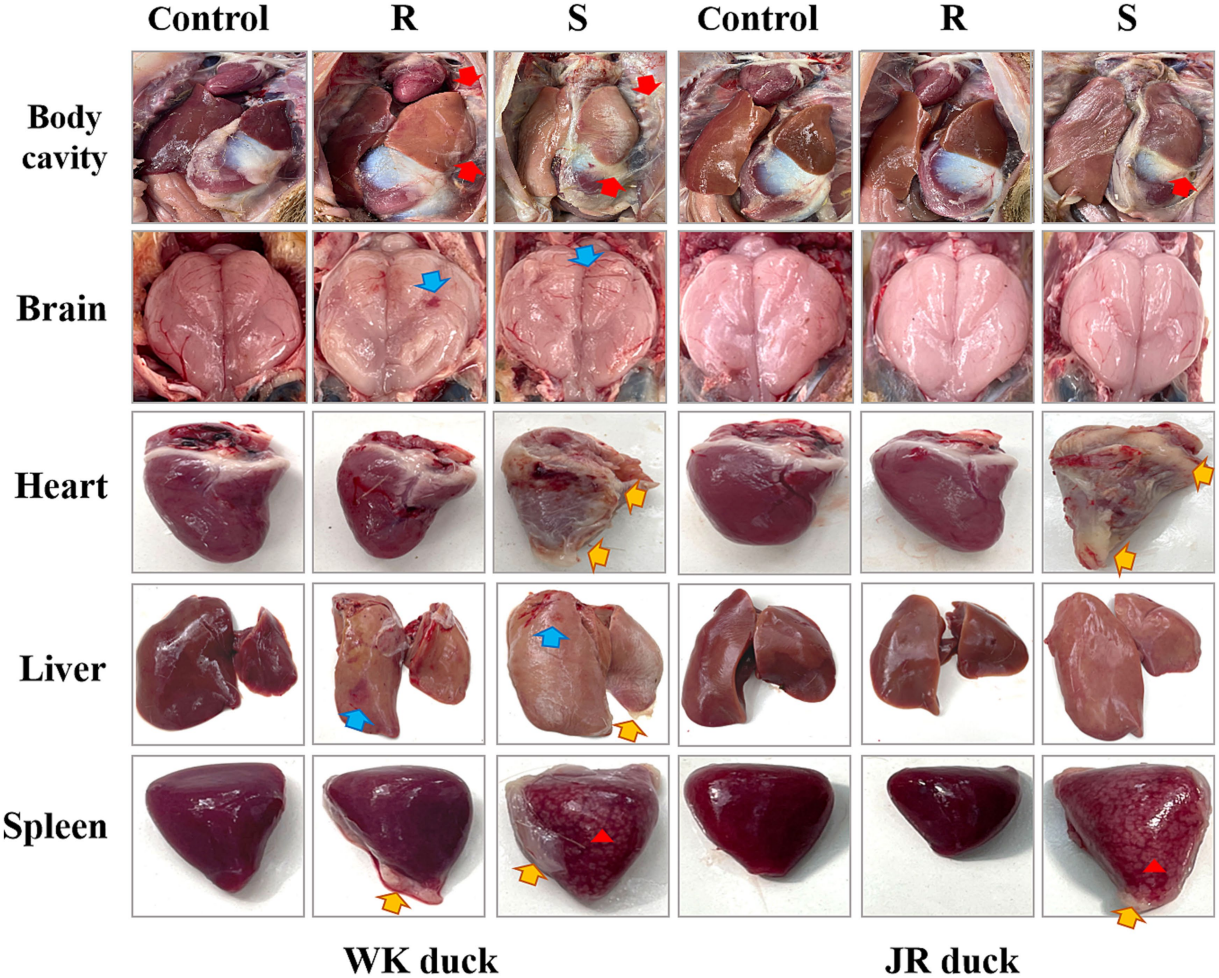
Gross pathologic lesions of ducklings with RA infection. Cellulose exudation (Red arrows). Inflammatory injury (Blue arrows). Cellulose pericarditis (Yellow arrows). Marbling lesions (Red triangle). Each group had at least three samples. Control: healthy control group. R: resistant group. S: susceptible group.

Using 72 hpi as the diagnostic endpoint, clinically asymptomatic subjects were designated as the disease-resistant group (R-group), while those presenting end-stage symptoms were classified as disease-susceptible group (S-group). Gross pathological analysis indicated that RA infection induced inflammatory injury of brain tissue (blue arrows), fibrinous pericarditis (yellow arrows) and marble lesions of the spleen (red triangle) in susceptible groups of both breeds (Figure 1). Notably, resistant WK ducks also displayed distinct tissue lesions, whereas resistant JR ducks maintained normal gross morphology. These findings substantiate the superior RA resistance exhibited by JR ducks compared to WK ducks across mortality, morbidity, and pathological parameters.

### 3.2 RA infection induces growth suppression and immune dysregulation

To assess the impact of RA infection on growth performance and immune function, body weight gain and spleen index were evaluated at 72 hpi. The results revealed significant inter- and intra-breed differences in JR and WK ducks. Inter-breed comparisons showed that the weight gain of control WK ducks was higher than that of control JR ducks; following infection, resistant WK ducks experienced a severe 76.8% reduction in weight gain relative to controls (*p* < 0.01), whereas resistant JR ducks maintained near-normal weight with only a 6.53% decrease (*p* > 0.05). Susceptible WK ducks exhibited a 174.4% reduction in weight gain versus controls, slightly less pronounced than susceptible JR ducks (185.5%) (Table 2). Intra-breed comparisons revealed resistant individuals in both breeds maintained significantly higher weights than susceptible counterparts (*p* < 0.01) (Table 2). Regarding immune organ metrics, both susceptible and resistant WK ducks had elevated spleen indices versus controls (*p* < 0.05), though resistant WK ducks displayed lower indices than susceptible WK ducks (*p* < 0.05) (Table 3). In contrast, JR ducks showed divergent responses: susceptible individuals showed increased spleen indices while resistant groups maintained baseline levels (Table 3), consistent with weight gain patterns. Collectively, RA infection induces growth impairment and immune dysregulation in a severity-dependent manner, with WK ducks showing greater overall vulnerability than JR ducks.

**Table 2:** Effects of RA infection on body weight of JR ducks and WK ducks.

Breed	Body weight gain (g)		
	Control-group	R-group	S-group
WK duck	132.2 ± 11.03^A^	30.7 ± 6.18^B^	−98.3 ± 7.10^C^
JR duck	106.67 ± 11.26^A^	99.7 ± 10.23^A^	−91.2 ± 5.98^B^

Different uppercase letters represent extremely significant differences (*p* < 0.01), and the same letters represent insignificant differences (*p* > 0.05). Each group had at least nine samples.

**Table 3:** Effects of RA infection on spleen index of JR ducks and WK ducks.

Breed	Spleen index (%)		
	Control-group	R-group	S-group
WK duck	0.093 ± 0.023^a^	0.126 ± 0.028^b^	0.237 ± 0.049^c^
JR duck	0.087 ± 0.0192^a^	0.092 ± 0.019^a^	0.173 ± 0.053^b^

Different lowercase letters represent significant differences (*p* < 0.05), and the same letters represent insignificant differences (*p* > 0.05). Each group had at nine samples.

### 3.3 RA infection induces systemic histopathological damage in ducks

**Figure 2:**
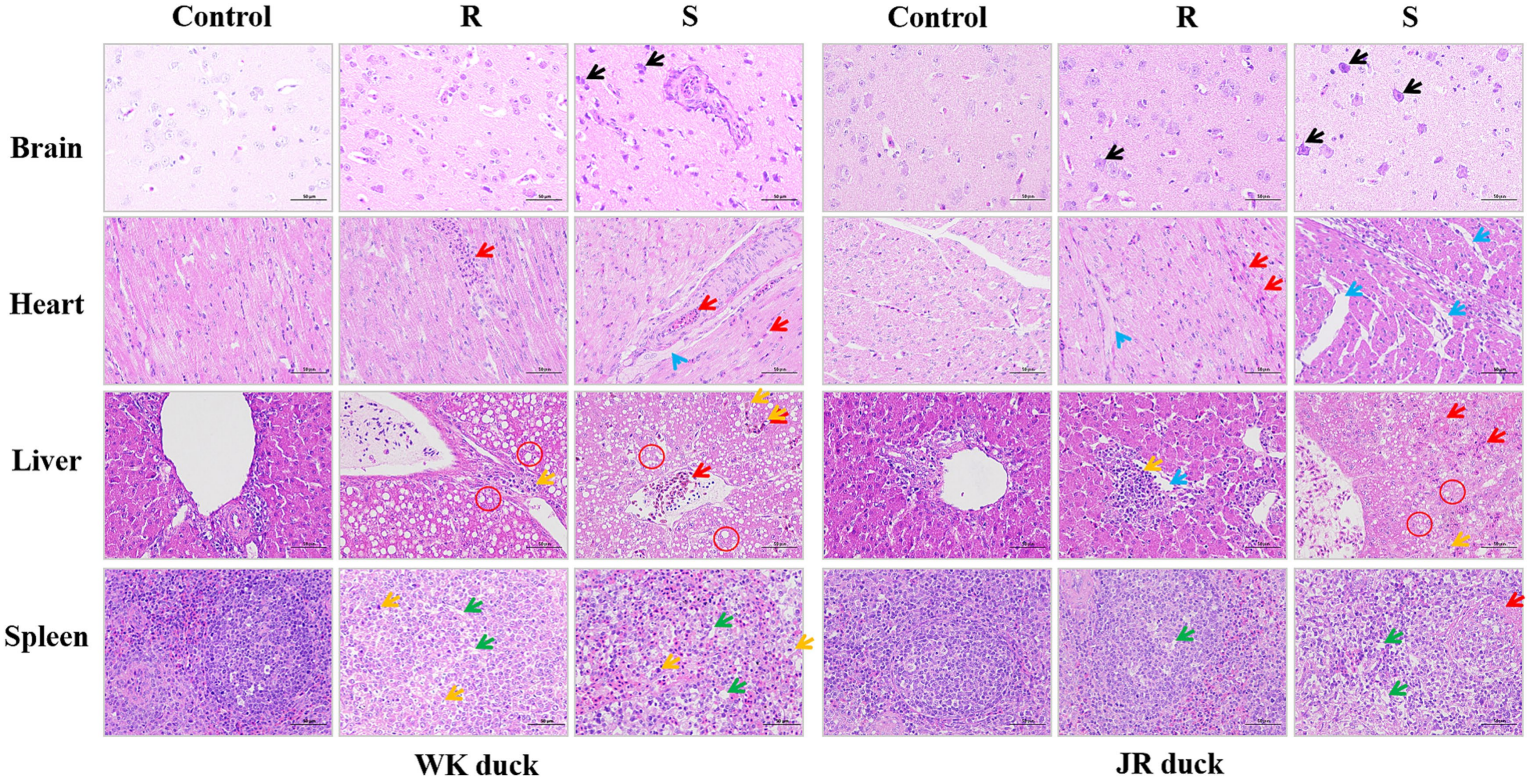
Effect of RA infection on histopathology of brain, heart, liver, and spleen in ducks. Nerve cell body shrinkage (Black arrows); Lymphocyte infiltration (Red arrows); interstitial edema (Blue arrows); Vacuolate-like degeneration (Red circle); Monocyte proliferation (Yellow arrows); Lymphocytes reduction (Green arrows). (Magnification: ×40; scale bar: 50 μm). Each group had at least three samples. Control: healthy control group. R: resistant group. S: susceptible group.

Histopathological analyses of the brain, heart, liver, and spleen of ducks revealed systemic histopathological damage following RA infection, with severity strongly correlated to disease susceptibility. As shown in Figure 2, the brain in the control group exhibited normal architecture with intact neuronal structure and no pathological changes. Following RA infection, both susceptible ducks in both breed exhibited severe neurodegeneration (black arrows) featuring perivascular lymphocyte cuffing, nuclear pyknosis, cytoplasmic eosinophilia, and satellitosis. Notably, the pathological changes in the resistant group were relatively less severe compared to the susceptible group, manifested as focal neuronal swelling and mild cytoplasmic eosinophilia without necrosis. There are no obvious differences among the varieties. Regarding the heart, the control group displayed organized myocardial fibers without inflammation or necrosis. In contrast, after RA infection, susceptible WK ducks showed focal myocardial necrosis with lytic fiber dissolution and dense lymphocytic infiltration (red arrows), while susceptible JR ducks exhibited interstitial edema (blue arrows) with cytoplasmic granular deposits and vacuolar degeneration; compared to the susceptible groups, the pathological changes in the resistant groups were relatively milder, mainly manifested as minor lymphocyte infiltration without structural damage in both breeds. Regarding the liver, following RA infection, susceptible ducks in both breed exhibited hepatic lobular architecture disruption, hepatocyte vacuolar degeneration (red circle), vascular congestion, and fibrinous exudation. In contrast, the liver of resistant ducks showed breed-specific pathological differences: WK ducks retained vacuolar degeneration and reticular structuring, while JR ducks displayed moderate inflammatory cell infiltration (yellow arrows) without vacuolar degeneration. Regarding the spleen, both breeds exhibited splenic cord disorganization and lymphoid depletion (green arrows) in susceptibles, with no interbreed differences. Taken together, these results indicate that RA infection caused significant multiorgan histopathological damage with severity directly correlates with host susceptibility status, showing organ-specific interbreed variation in cardiac and hepatic responses.

### 3.4 Effects of RA infection on humoral immunity of ducks

**Figure 3:**
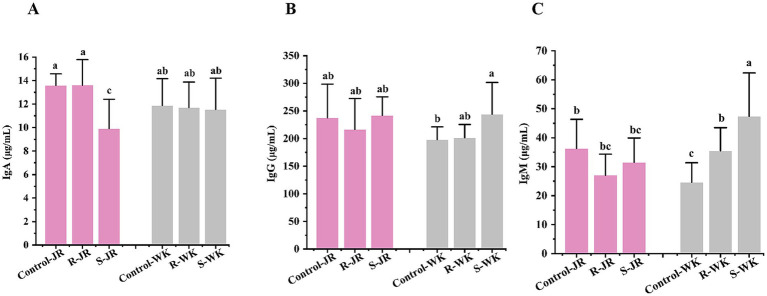
Effects of RA infection on humoral immunity of ducks. **(A)** Serum level of IgA after RA infection. **(B)** Serum level of IgG after RA infection. **(C)** Serum level of IgM after RA infection. Each group had at least five samples. Bars with identical letters indicate no significant difference (*p* > 0.05), while bars with different letters indicate significant difference (*p* < 0.05). Control-JR: healthy control group in JR ducks. R-JR: resistant group in JR ducks. S-JR: susceptible group in JR ducks. R-WK: resistant group in WK ducks. S-WK: susceptible group in WK ducks. Control-WK: healthy control group in WK ducks.

To evaluate the role of humoral immunity in defense against RA infection, serum IgA, IgG, and IgM levels were quantified via ELISA in RA-infected ducks. The results revealed significant differences both within and between the JR and WK breeds (Figure 3). Inter-breed analysis showed that susceptible WK ducks displayed elevated IgM and IgG levels (Figures 3B,C) with stable IgA (Figure 3A). Conversely, susceptible JR ducks exhibited significant IgA downregulation compared to controls (*p* < 0.05) (Figure 3A), while their IgG and IgM levels remained unchanged (*p* > 0.05) (Figures 3B,C). In contrast, susceptible WK ducks exhibited elevated IgM and IgG levels while maintaing a stable IgA level. In contrast, resistant WK ducks showed a significant increase in IgM (though lower than in susceptible WK ducks) alongside stable IgG and IgA (*p* < 0.05), whereas resistant JR ducks mantained sable IgA, IgM, and IgG levels (*p* > 0.05). Intra-breed comparisons revealed that resistant WK ducks displayed lower IgM and IgM levels than that of susceptible counterparts (*p* < 0.05), whereas resistant JR ducks showed higher IgA level than that of susceptible counterparts (*p* < 0.05). These results suggest distinct humoral immune stategies: JR ducks stimulate a response dominated by IgA, whereas WK ducks activate a response dominated by IgG and IgM.

### 3.5 Effects of RA infection on Th1/Th2/Th17 cytokines response in ducks

**Figure 4:**
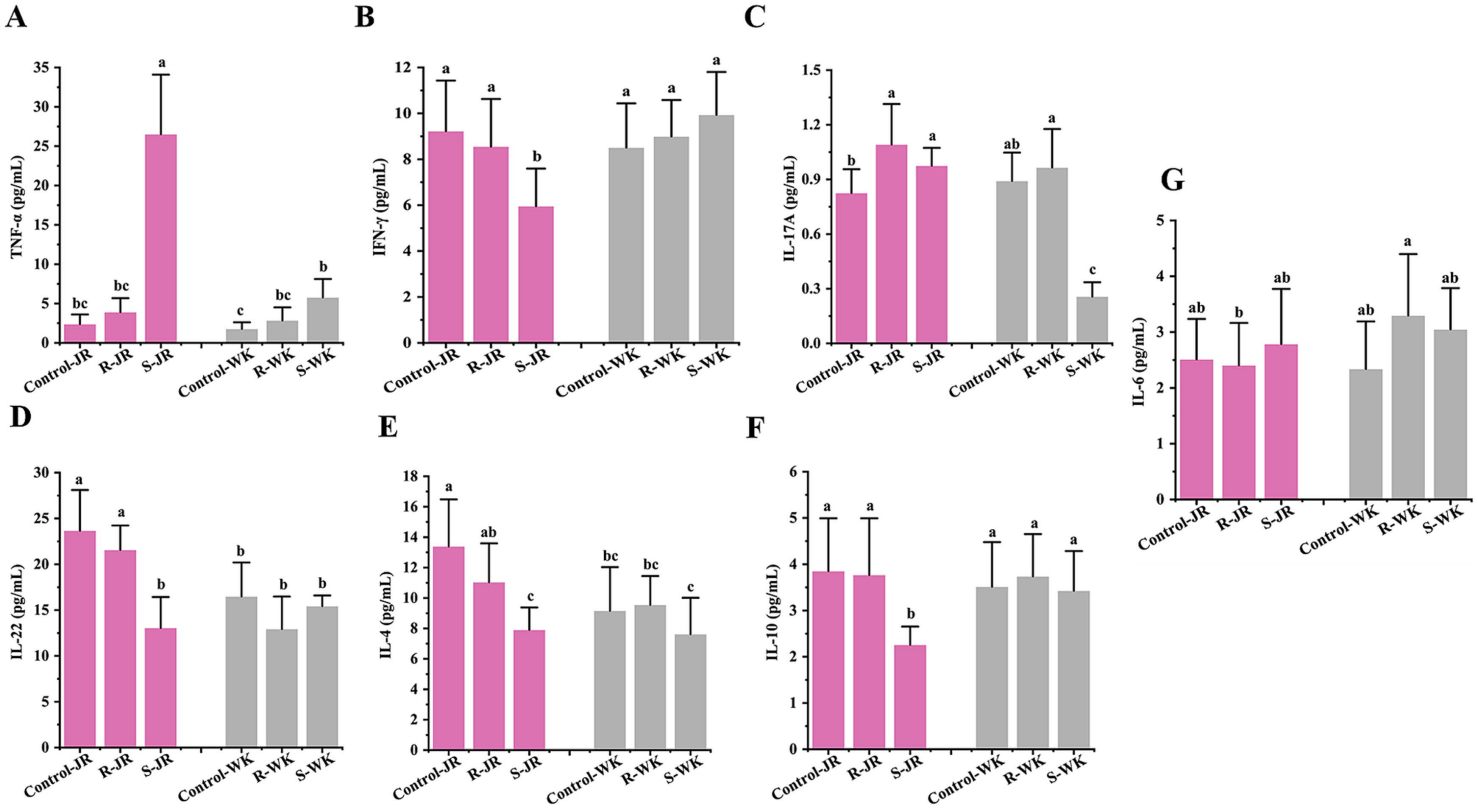
Effects of RA infection on Th1/Th2/Th17 cytokines response in ducks by ELISA. **(A,B)** Serum level of Th1 markers TNF-*α* **(A)** and IFN-*γ* **(B)** after RA infection. **(C,D)** Serum level of Th17 markers IL-17A **(C)** and IL-22 **(D)** after RA infection. **(E,F)** Serum level of Th2 markers IL-4 **(E)** and IL-10 **(F)** after RA infection. **(G)** Serum level of IL-6 after RA infection. Each group had at least five samples. Bars with identical letters indicate no significant difference (*p* > 0.05), while bars with different letters indicate significant difference (*p* < 0.05). Control-JR: healthy control group in JR ducks. R-JR: resistant group in JR ducks. S-JR: Susceptible group in JR ducks. R-WK: resistant group in WK ducks. S-WK: Susceptible group in WK ducks. Control-WK: healthy control group in WK ducks.

Cytokine response is an important part of host innate immune system. Evaluation of cytokine responses via ELISA following RA infection revealed distinct patterns both within and between the JR and WK breeds. Inter-breed comparison revealed that susceptible WK ducks exhibited significant elevation of TNF-*α* levels (*p* < 0.05) (Figure 4A) with concomitant suppression of IL-17A (*p* < 0.05) (Figure 4C), while IFN-*γ* (Figure 4B), IL-22 (Figure 4D), IL-4 (Figure 4E), and IL-10 (Figure 4F) remained statistically unchanged relative to control group (*p* > 0.05). Conversely, susceptible JR ducks exhibited broad suppression of IFN-*γ*, IL-22, IL-4, and IL-10 (*p* < 0.05) alongside TNF-*α* elevation (Figure 4G), with IL-17A stable (*p* > 0.05); notably, susceptible JR ducks exhibited higher TNF-*α* level than susceptible WK ducks despite similar basal expression. In contrast, resistant WK ducks demonstrated significant TNF-*α* upregulation (*p* < 0.05) without other cytokine alterations (*p* > 0.05), whereas resistant JR ducks uniquely showed elevated IL-17A (*p* < 0.05) with all other mediators unchanged (*p* > 0.05). Both breeds showed no significant change in expression level of IL-6 after RA infection (*p* > 0.05) (Figure 4G). Intra-breed analysis showed that susceptible JR ducks had higher levels of IFN-*γ*, IL-22, IL-4, and IL-10 but lower TNF-*α* level compared to resistant counterparts and controls, while susceptible WK ducks showed higher TNF-*α* level and lower IL-17A level relative to resistant ducks and controls. These results demonstrate breed-specific cytokine response strategies to RA infection.

### 3.6 RA infection induces Iron metabolism dysregulation in ducks

**Figure 5:**
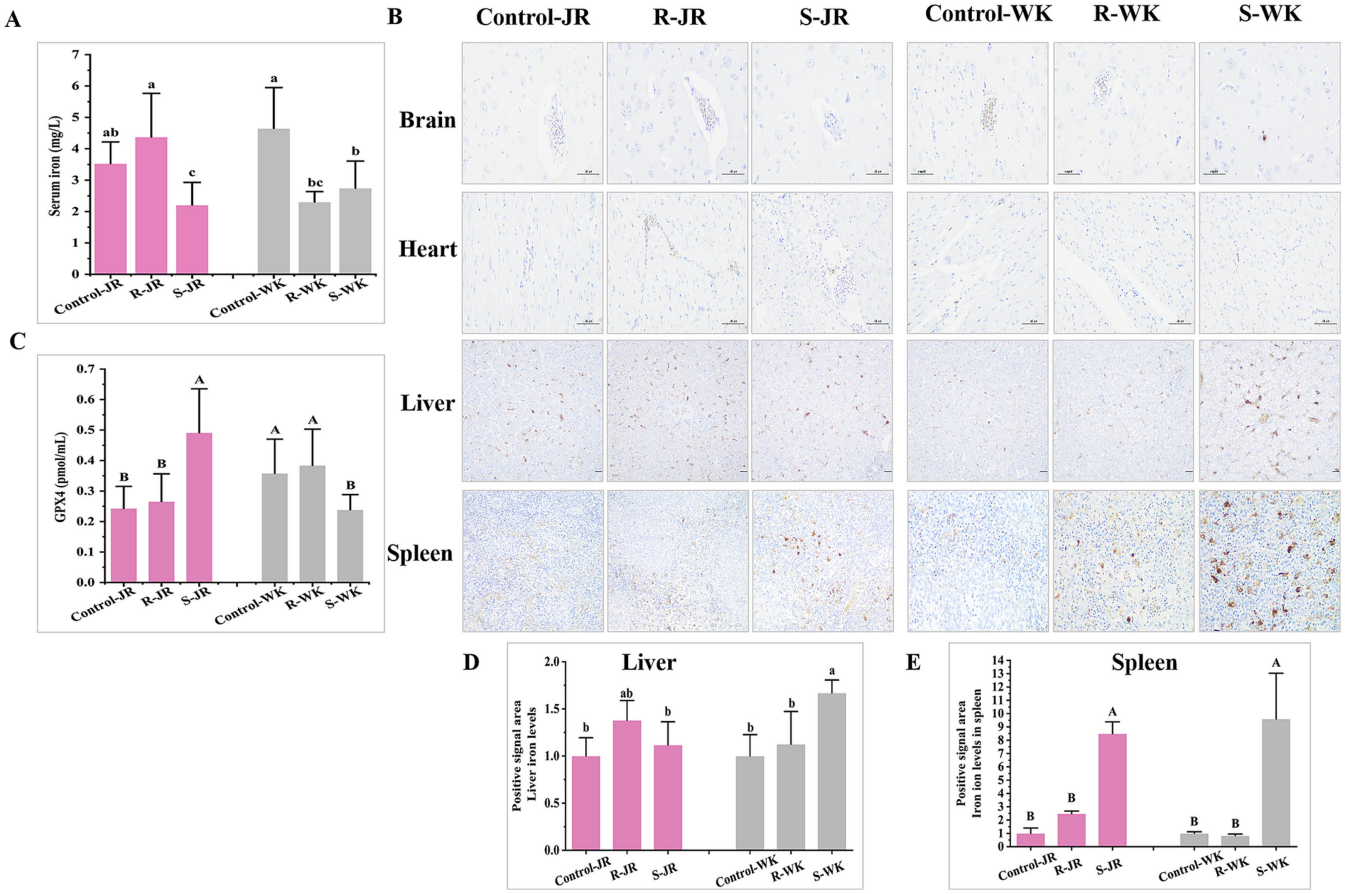
Effects of RA infection on iron metabolisn in ducks. **(A)** Content of iron in serum of ducks infected with RA by ELISA. **(B)** Iron deposition levels in the brain, heart, and livers infected with RA by Prussian blue staining (Magnification: ×20; scale bar: 100 μm). **(C)** Expression level of GPX4 in serum of ducks infected with RA by ELISA. **(D)** Quantitative analysis of iron deposition levels in the liver of ducks infected with RA via ImageJ. **(E)** Quantitative analysis of iron deposition levels in the spleen of ducks infected with RA via ImageJ. Bars with identical letters indicate no significant difference (*p* > 0.05), while bars with different letters indicate significant difference (*p* < 0.05). Each group had at least three samples. Control-JR: healthy control group in JR ducks. R-JR: resistant group in JR ducks. S-JR: susceptible group in JR ducks. R-WK: resistant group in WK ducks. S-WK: susceptible group in WK ducks. Control-WK: healthy control group in WK ducks.

As a key mediator of innate immunity, iron plays an important role in RA infection. Hepatic and splenic iron overload, coupled with glutathione peroxidase 4 (GPX4) downregulation, was linked to ferroptosis. Evaluation of iron metabolism via ELISA following RA infection revealed distinct patterns both within and between the JR and WK breeds. Inter-breed comparison indicated that WK ducks developed hypoferremia in both susceptible and resistant groups compared to uninfected controls (*p* < 0.05) (Figures 5A,C), with susceptible individuals showing pronounced GPX4 downregulation (*p* < 0.01) (Figure 5A). Conversely, susceptible JR ducks exhibited hypoferremia but paradoxically displayed GPX4 upregulation (*p* < 0.05), while resistant ducks maintained physiological serum iron homeostasis (Figure 5A). Results of Prussian blue staining analysis revealed susceptible WK ducks showed marked iron accumulation in the liver (+1.67-fold, *p* < 0.05) (Figures 5B,D) and spleen (+9.60-fold, *p* < 0.001) (Figures 5B,E) versus controls, while resistant ducks retained baseline iron levels. In contrat, susceptibles JR ducks exhibited spleen-specific iron overload (8.49-fold, *p* < 0.05) (Figures 5B,E) without hepatic deposition (Figures 5B,D), while resistant ducks maintanied stable iron level in both organs. Notably, baseline iron concentrations showed no interbreed differences between healthy WK and JR ducks prior to infection (*p* > 0.05). No pathological iron deposition was observed in neural or cardiac tissues across groups. These results underscore iron metabolism as a critical determinant of RA infection outcomes, highlighting divergent host adaptation strategies between duck breeds.

### 3.7 Correlation analysis of immune indexes induced by RA infection

**Figure 6:**
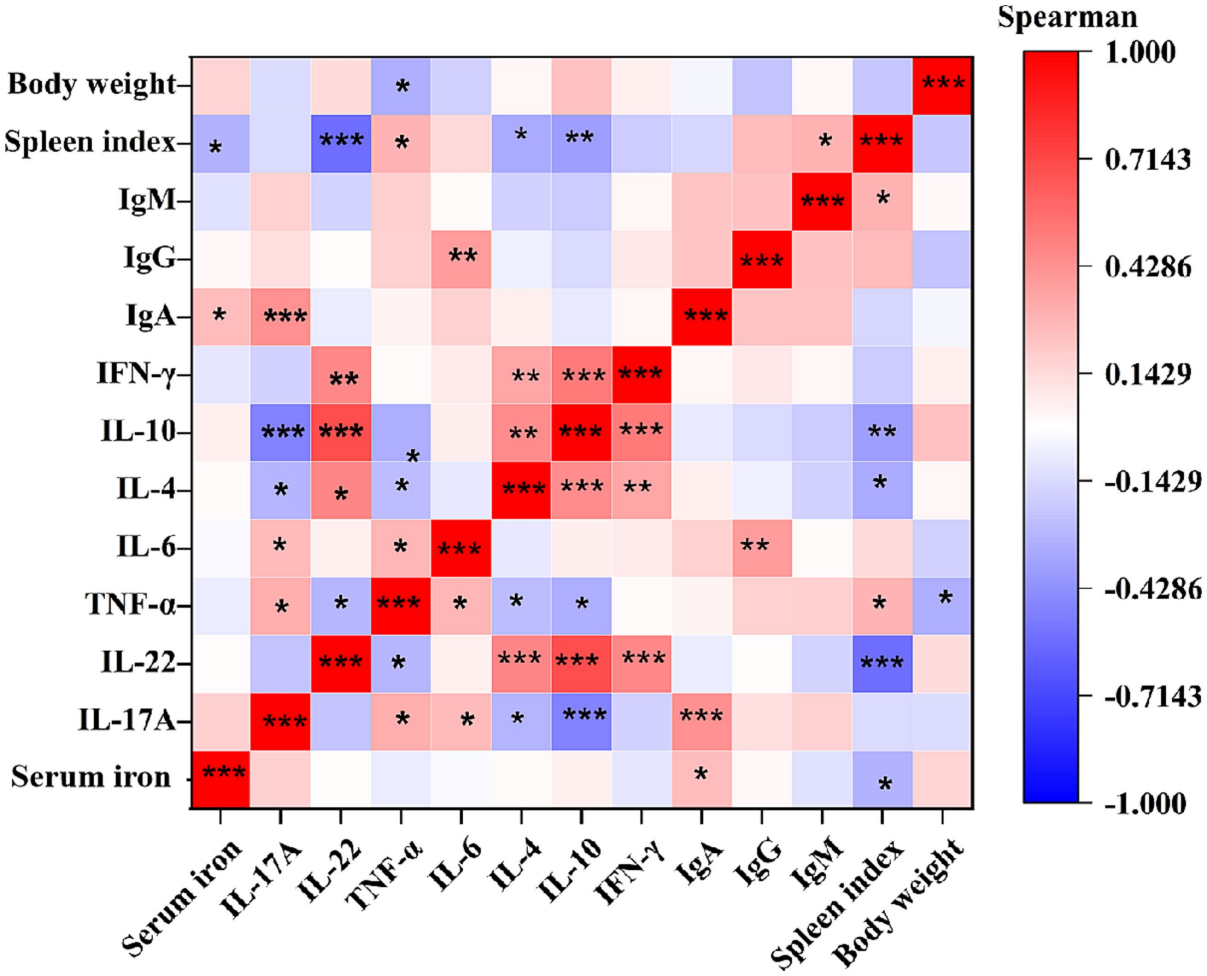
Spearman correlation analysis of immune indexes induced by RA infection. *r* represents Spearman correlation coefficient. *Indicates significant difference (*p* < 0.05); **indicates significant difference (*p* < 0.01); ***indicates significant difference (*p* < 0.001). Each group had at least nine samples.

Spearman correlation analysis was conducted to investigate the interplay between body weight gain, spleen index, humoral immunity, cytokine networks, and iron-mediated immune responses. As shown in Figure 6, the results revealed a modest but significant negative correlation between body weight gain and TNF-*α* level (*r* = −0.32, *p* = 0.0144). The spleen index exhibited negative correlations with serum iron content (*r* = −0.30, *p* = 0.0211), IL-22 (*r* = −0.56, *p* = 5.02E-06), (*r* = −0.30, *p* = 0.0211), IL-4 (*r* = −0.33, *p* = 0.01216), and IL-10 (*r* = −0.38, *p* = 0.0037), along with weaker positive associations with TNF-*α* (*r* = 0.29, *p* = 0.0259), and IgM (*r* = 0.30, *p* = 0.02215). In contrast, IgA displayed positive correlations with serum iron (*r* = 0.26, *p* = 0.04508) and IL-17A (*r* = 0.44, *p* = 5.27E-04), while IgG showed a positive correlation with IL-6 (*r* = 0.38, *p* = 0.00262). Regarding cytokines, pro-inflammatory cytokines IL-17A, TNF-*α*, and IL-6 were mutually positively correlated (*r* range: 0.27–0.31 *p* < 0.05), while IL-4, IL-10, IL-22, and IFN-*γ* a tightly interconnected positive cluster (*r* rang: 0.45–0.69 *p* < 0.01). Notably, IL-17A showed significant inverse relationships with Th2 cytokines IL-4 (*r* = −0.29, *p* = 0.0251) and IL-10 (*r* = −0.49, *p* = 9.23E-05), but no association with IL-22 (*p* > 0.05). TNF-*α* exhibited modest negative correlations with IL-4 (*r* = −0.26, *p* = 0.0444), IL-10 (*r* = −0.31698, *p* = 0.01444), and IL-22 (*r* = −0.28568, *p* = 0.02829). These findings delineate a multi-layered immune strategy in ducks resisting RA infection, integrating humoral immunity, inflammatory regulation, and iron metabolism.

## 4 Discussion

This study delineates divergent immune-iron adaptation strategies to RA infection between JR and WK duck breeds, and between resistant and susceptible phenotypes within breeds. Inter-breed comparison results indicate that JR ducks exhibited lower mortality and morbidity compared to WK ducks under RA infection; while susceptible individuals in both breeds showed severe weight loss, splenomegaly, and multiorgan histopathology, and resilience mechanisms diverged critically: resistant JR ducks maintained stable growth, normal spleen indices, minimal lesions, physiological iron homeostasis, and mounted an IL-17A-elevated response with stable IgA/Th1/Th2 cytokines. Conversely, resistant WK ducks still exhibited significant weight loss, splenomegaly, histopathological damage, systemic iron accumulation, and TNF*α*-dominated immunity with reduced IgM. These trends align with reports that indigenous breeds may retain robust innate resistance traits potentially diluted in commercial breeds during artificial selection (Weerasooriya et al., 2017; Li et al., 2018; Wang et al., 2022; Li et al., 2025).

Humoral immunity plays crucial roles in combating bacterial infections, with IgM, IgG, and IgA antibodie serving as key effectors against RA infection (Higgins et al., 2000; Zhang et al., 2014; Kang et al., 2018; Eladl et al., 2023). IgM is the first antibody to respond after infection. It rapidly binds to bacterial surface antigens to initiate immune reactions but declines as IgG and IgA become more prominent (Magor, 2011; Keyt et al., 2020; Aldayel et al., 2025). IgG dominates in the later stages, providing sustained protection through high-affinity antigen neutralization. IgA, the most abundantly produced antibody in mammals, plays a critical role in mucosal immunity. Mucosal IgA safeguards the integrity of the epithelial barrier by preventing pathogen colonization, invasion, and dissemination, a function particularly critical for defending against enteric infections. A previous study revealed that RA infection could disrupt the intestinal barrier structure of the duck’s cecum, triggering both inflammatory and mucosal immune response predominantly during the early to mid-stages (1–9 hpi) (Tao et al., 2020). Consistent with these findings, secreted IgA has been identified as a primary immune effector against RA, with its concentration directly correlating to host protective capacity (Kang et al., 2018). In our study, humoral profiling revealed WK ducks’ severity-dependent IgM/IgG upregulation versus JR ducks’ phenotype-specific IgA regulation after RA infection, where stable IgA in resistant JR ducks likely optimizes mucosal barrier protection against RA’s intestinal/respiratory invasion (Gleeson et al., 2024), aligning with IgA’s dual role in pathogen exclusion and inflammation control (Pabst, 2012). By modulating circulating IgA levels, JR ducks likely optimize mucosal barrier protection while minimizing collateral tissue damage, a strategy potentially triggered by RA’s invasion of the intestinal and respiratory mucosa (Tao et al., 2020).

Cytokines serve as pivotal regulators of immune and inflammatory responses during bacterial infections. Pro-inflammatory cytokines IFN-*γ* are related to the Th1 immune response, which is mainly participated in cell-mediated defenses and inflammatory activation (Yang et al., 2019; Tang et al., 2021). Anti-inflammatory cytokines IL-4 and IL-10 are related to the Th2 immune response, which is primarily associated with humoral immune responses and tissue repair (Yang et al., 2019). IL-17A and IL-22 are related to Th17 immune response, which is mainly involved in mucosal immunity and plays critical roles in resisting pathogen infection. Th2 cytokines can inhibit Th1/Th17 cell differentiation and function to avoid excessive cellular immune response to the body damage (Fernandez et al., 2018). In the present study, cytokine network analysis revealed distinct patterns both between JR and WK breeds and within each breed. Between breeds, susceptible WK ducks exhibited exhibited a stable Th1/Th2 axis (defined by IL-4, IL-10, IFN-*γ*) alongside increased TNF-*α* and decreased IL-17A, whereas susceptible JR ducks displayed profound suppression of the Th1/Th2 axis with concurrent hyperactivation of TNF-*α* and IL-17A. Within breeds, susceptible WK ducks maintained this stable Th1/Th2 axis but with altered TNF-α/IL-17A levels (elevated TNF-*α*, reduced IL-17A) compared to their resistant counterparts, which showed a stable overall Th1/Th2/Th17 axis. Within the JR breed, susceptible individuals exhibited the aforementioned Th1/Th2 suppression and TNF-*α*/IL-17A hyperactivation, contrasting with resistant JR ducks that maintained a stable Th1/Th2 axis alongside elevated IL-17A levels. Given the balance between Th1/Th2/Th17 cytokines is critical to the cell’s defense against RA infection (Liu et al., 2013; Zhang et al., 2014; Fernandez et al., 2016; Fernandez et al., 2018; Wu et al., 2020). Further correlation analysis showed that TNF-*α,* which was correlated with splenomegaly, was significantly negatively correlated with Th2 cytokines and body weight gain (Figure 6). These findings suggest that the downregulation of Th1/Th2 coupled with TNF-*α* overexpression may be associated with the susceptibility of susceptible JR ducks, whereas the stable expression of Th1/Th2 alongside TNF-*α* upregulation and Th17 suppression might be linked to the susceptibility of susceptible WK ducks. As IL-17A was positively linked to IgA while negatively correlating with disease severity markers (Figure 6), consistent with previous reports that IL-17A promoted protective IgA responses (Dann et al., 2015; Karaffova et al., 2015; Watorek and Klinger, 2015; Liao et al., 2022). These findings indicate that resistant JR ducks may deploy a multi-layered defense including: i IL-17A-driven mucosal immunity synergizes with IgA to fortify barrier function (Karaffova et al., 2015; Zhang et al., 2022); ii preserved Th1/Th2 balance prevents inflammatory escalation (Zhao et al., 2020); ii controlled TNF-*α* activity mitigates tissue damage. Taken together, the coordinated IL-17A-IgA axis in resistant JR ducks may contribute to clinical outcomes, whereas dysregulated TNF-*α* in WK ducks could exacerbate pathology. The molecular pathways underlying the Th17-IgA coordination in JR ducks remains to be further studied.

Iron serves as a dual-edged sword in host-pathogen dynamics, balancing microbial survival and host defense (Muckenthaler et al., 2017). RA exploits iron for virulence and invasion, while hosts counter with iron restriction-an evolutionarily conserved strategy (Cassat and Skaar, 2013; Tu et al., 2014; Wang et al., 2025). However, iron overload triggers Fenton reactions, driving ferroptosis via lipid peroxidation and oxidative cascades (Webster and Guerinot, 2024). Glutathione peroxidase 4 (GPX4) is a key lipid peroxidase that inhibits ferroptosis by catalyzing glutathione (GSH) to eliminate lipid peroxides, thus preventing lipid peroxidation in cell membranes and maintaining membrane stability (Yang and Stockwell, 2016; Stockwell et al., 2017). GPX4 inactivation disrupts antioxidant defenses, impairs cellular clearance of lipid peroxides, and triggers iron-dependent lipid peroxidation chain reactions, ultimately inducing cellular ferroptosis (Chen et al., 2025; Huang et al., 2025). In this study, WK ducks developed hypoferremia in both susceptible and resistant groups after RA infection coincided with hepatic/splenic iron overload and perihepatic inflammation (Figures 5A,D,E), yet GPX4 remained static or downregulated (Figure 5B). Though direct causal links require validation, the association between iron accumulation and tissue damage in WK ducks suggests impaired iron handling may compound pathology. This aligns with recent findings in Salmonella infected- macrophages, where iron overload and GPX4 inhibition drive ferroptosis (Liu et al., 2025; Sui et al., 2025). Conversely, JR ducks displayed phenotype-dependent regulation-susceptibles developed hypoferremia with GPX4 upregulation, while resistants maintained hyperferremia and physiological iron in liver/spleen. The liver and spleen, as dual hubs of iron storage and immunity, emerged as critical battlegrounds. Susceptible WK ducks’ failure to upregulate GPX4 likely exacerbated ferroptosis, evidenced by splenic apoptosis and systemic iron dysregulation. In contrast, JR ducks’ compartmentalized iron handling-splenic iron retention in susceptibles and systemic equilibrium in resistants-coupled with GPX4 plasticity, highlights an adaptive mechanism to balance pathogen starvation (via hypoferremia) and oxidative protection.

While this study reveals phenotypic and molecular trends distinguishing duck breeds’ responses to RA infection, key limitations warrant further investigation: (i) Conduct *in vivo* RA challenge studies with IL-17A knockout/JR ducks and TNF-*α* inhibitors in WK ducks to functionally validate observed immune patterns; (ii) perform transcriptomics and metabolomics analyses to identify regulatory networks underlying IgA/Th17 maintenance and iron metabolism in JR ducks; (iii) explore whether iron chelators or ferroptosis inhibitors ameliorate tissue damage in infected WK ducks; (iv) establish F2 intercross populations to map QTLs associated with mucosal immunity stability and iron regulation capacity.

## 5 Conclusion

This study demonstrates that JR ducks have stronger natural resistance to RA infection compared to WK ducks in terms of mortality, morbidity, tissue lesions, immunoglobulin and cytokine responses, and iron metabolism regulation, antibody production, and iron metabolism. These findings provide important insights into the resistance mechanisms of ducks to RA infection and valuable references for the development of disease-resistant waterfowl breeding and disease-control strategies.

## Data Availability

The original contributions presented in the study are included in the article/supplementary material, further inquiries can be directed to the corresponding author.
